# Modulation of Macrophage Polarization by Viruses: Turning Off/On Host Antiviral Responses

**DOI:** 10.3389/fmicb.2022.839585

**Published:** 2022-02-11

**Authors:** Shaoxiong Yu, Hailiang Ge, Su Li, Hua-Ji Qiu

**Affiliations:** State Key Laboratory of Veterinary Biotechnology, National African Swine Fever Para-Reference Laboratory, Harbin Veterinary Research Institute, Chinese Academy of Agricultural Sciences, Harbin, China

**Keywords:** macrophages, macrophage polarization, regulation network, antiviral responses, immune evasion

## Abstract

Macrophages are professional antigen-presenting cells and serve as the first line of defense against invading pathogens. Macrophages are polarized toward the proinflammatory classical (M1) or anti-inflammatory alternative (M2) phenotype upon viral infections. M1-polarized macrophages exert critical roles in antiviral responses *via* different mechanisms. Within the long competitive history between viruses and hosts, viruses have evolved various immune evasion strategies, inhibiting macrophage acquisition of an antiviral phenotype, impairing the antiviral responses of activated macrophages, and/or exploiting macrophage phenotypes for efficient replication. This review focuses on the sophisticated regulation of macrophage polarization utilized by viruses and is expected to provide systematic insights into the regulatory mechanisms of macrophage polarization by viruses and further facilitate the design of therapeutic targets for antivirals.

## Introduction

In the battle against viruses, immune cells, including macrophages, are essential fighters that directly kill viruses or secrete antiviral factors. Macrophages originate from bone marrow-derived monocytes. Upon inflammation, circulating monocytes in peripheral blood migrate into different tissues and are differentiated into several types of macrophages, such as microglia (central nervous system), alveolar macrophages (lung), Kupffer cells (liver), histocytes (spleen), and osteoclasts (bone marrow) (Epelman et al., 2014; Shapouri-Moghaddam et al., 2018).

Macrophages are multifunctional immune cells that exert their functions through phagocytosis, antigen presentation, and cytokine production. Macrophage activation is also known as polarization (Sang et al., 2015). Macrophages can be activated by diverse stimuli and signals and polarized into one of two phenotypes: the classical (M1) and alternative (M2) phenotypes (Murray, 2017; Shapouri-Moghaddam et al., 2018). M1 macrophages are characterized by the release of proinflammatory cytokines, while high-level anti-inflammatory cytokines are produced in M2 macrophages that are involved in tissue remodeling and repair. Macrophages can also be activated and polarized into the M1 or M2 phenotype in response to viral infections. M1-polarized macrophages are usually considered antiviral, while M2-polarized macrophages are considered immunosuppressive. Viruses have evolved multiple strategies to counteract the antiviral responses elicited by M1 macrophages and take advantage of M2-polarized macrophages for efficient replication (Sang et al., 2015).

This review presents an overview of the sophisticated regulation of macrophage polarization and focuses on the multiple immune evasion and exploitation mechanisms leveraged by various viruses against the antiviral responses of polarized macrophages.

## Macrophage Polarization Is a Delicately Regulated Cellular Process

### Induction of Macrophage Polarization

Upon stimulation, macrophages are differentiated into two distinct subpopulations, classical or inflammatory M1 macrophages and alternative or anti-inflammatory M2 macrophages (Shapouri-Moghaddam et al., 2018). M1 macrophages differentiation is induced by Th1 cytokines, such as interferon γ (IFN-γ) and interleukin 1β (IL-1β), or lipopolysaccharides (LPSs), and these macrophages produce several proinflammatory cytokines, including tumor necrosis factor α (TNF-α), interleukin 1α (IL-1α), IL-1β, IL-6, IL-12, and IL-23. Macrophage polarization into the M1 phenotype results in the increased expression of several marker molecules (such as CD80, CD86, and CD68), major histocompatibility complex class II (MHC-II), and inducible nitric oxide synthase 2 (iNOS2). In contrast, M2 macrophages are usually more sophisticated and are classified into four subtypes depending on the stimuli: M2a, M2b, M2c, and M2d (Wang et al., 2019). M2a macrophages are induced by IL-4 or IL-13 and characterized by high expression of the CD206 decoy receptor IL-1 receptor 2 (IL-1R2) or arginase 1 (Arg-1) and secretion of cytokines that contribute to tissue repair, such as transforming growth factor β (TGF-β) and IL-10. M2b macrophages are stimulated by immune complexes, Toll-like receptor (TLR) ligands or IL-1β and secret proinflammatory and anti-inflammatory cytokines, such as TNF-α, IL-1β, IL-6, IL-10, and CCL1. M2c macrophages are induced by IL-10, TGF-β, or glucocorticoids and characterized by the expression of CD163 and CD206 and exhibit anti-inflammatory activities by producing IL-10 and TGF-β. M2d macrophages are activated by TLR ligands and adenosine receptor ligands and characterized by the production of IL-10 and VEGF, which promote angiogenesis and tumor progression.

### Signaling Pathways in the Regulation of Macrophage Polarization

**FIGURE 1:**
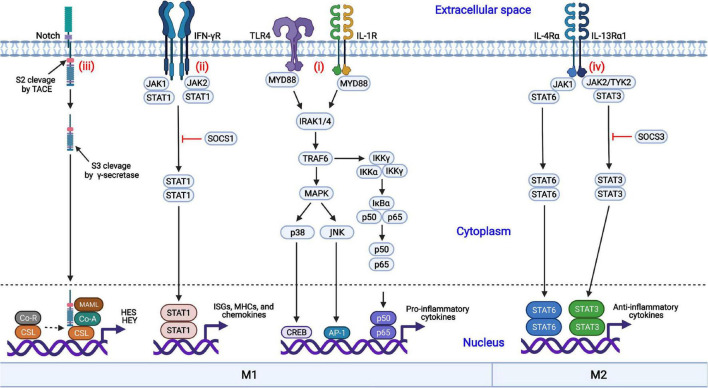
Coordinated regulation of macrophage polarization *via* multiple signaling pathways. (i) Engagement of TLR4 or IL-1R with the ligands results in the activation of the MAPK and NF-κB signaling pathways and induces the nuclear translocation of several transcription factors, promoting the production of proinflammatory cytokines; (ii) IFN-γ binds to its receptor, IFN-γR, and triggers the phosphorylation and dimerization of STAT1, initiating the transcription of IFN-stimulated genes (ISGs); (iii) The interactions of Notch proteins with Delta-like ligands and Jagged ligands induce the activation and nuclear translocation of the Notch intracellular domain (NICD), driving the production of proinflammatory cytokines; (iv) IL-4 or IL-13 binds to its corresponding receptor and triggers the activation of STAT3 or STAT6, respectively, inducing the transcription of anti-inflammatory cytokines and skewing macrophages to the anti-inflammatory M2 phenotype.

Macrophages are regulated by diverse coordinated signaling pathways, differentiated into the M1 or M2 phenotype, and exert multiple functions. The nuclear factor kappa B (NF-κB), Janus kinase/signal transducer and activator of transcription (JAK-STAT), mitogen-activated protein kinase (MAPK), and Notch signaling pathways are involved in macrophage polarization (Figure 1).

#### Nuclear Factor Kappa B Signaling Pathway

NF-κB is an important transcription factor involved in the production of proinflammatory cytokines. The NF-κB signaling pathway is activated by TLRs (such as TLR4) or cytokine receptors [such as the IL-1 receptor (IL-1R)] in response to their ligands (LPSs and IL-1, respectively), resulting in the activation of the IκB kinase (IKK) complex (Liu et al., 2014; Dorrington and Fraser, 2019). Then, IκBα is phosphorylated by the activated IKK complex and quickly undergoes ubiquitination followed by proteasomal degradation (Mulero et al., 2019). The NF-κB dimers p50/p65 are released from IκBα, are translocated into the nucleus and initiate the production of inflammatory cytokines, such as IL-1β, IL-6, and TNF-α, which result in M1 macrophage polarization (Biswas and Lewis, 2010).

#### Janus Kinase/Signal Transducer and Activator of Transcription Signaling Pathway

The JAK-STAT signaling pathway is involved in both M1 and M2 macrophage polarization. IFN-γ interacts with the IFN-γ receptor (IFN-γR), activates the receptor-associated tyrosine kinases JAK1 and JAK2, and subsequently promotes the phosphorylation and dimerization of STAT1 (Ivashkiv, 2018). Activated STAT1 homodimers are translocated into the nucleus, bind to the IFN-γ-activation sites (GASs), and induce the transcription of interferon-stimulated genes (ISGs), MHC molecules, chemokines, and antiviral factors, promoting macrophage polarization toward the M1 phenotype. In contrast, STAT3 or STAT6 homodimers are translocated into the nucleus in response to IL-4 or IL-13 treatment, resulting in the transcription of the genes encoding M2 phenotype-related anti-inflammatory cytokines, such as TGF-β and IL-10. In addition, suppressor of cytokine signaling 1 (SOCS1) and SOCS3 block the activation of the STAT1- and STAT3-mediated signaling pathways, respectively.

#### Mitogen-Activated Protein Kinase Signaling Pathway

In addition to the NF-κB signaling pathway, the MAPK signaling pathway is also crucial to proinflammatory cytokine production (Arthur and Ley, 2013). The activation of MAPK by TLRs is a well-characterized signaling pathway. Following the activation of TLR4 by its cognate ligands, the signals are transduced through the cytoplasmic Toll and IL-1 receptor (TIR) domain, recruiting myeloid differentiation primary response protein 88 (MYD88) with the requirement of MYD88 adaptor-like protein (TIRAP). IL-1 receptor-associated kinase 4 (IRAK4) interacts with MYD88 and induces the formation of a complex comprised of IRAK1, IRAK2, and TNF receptor-associated factor 6 (TRAF6). Then, TRAF6 undergoes self-ubiquitination by E2 ubiquitin-conjugating enzyme 13 (UBC13) through K63-linked ubiquitin chains. Following the recruitment of TGF-β-activated kinase 1 (TAK1) to TRAF6 by TAK1-binding protein 2 (TAB2) and TAB3, p38α and Jun N-terminal kinase (JNK) are activated and induce the expression of proinflammatory cytokines, promoting M1 macrophage polarization (Yang et al., 2014).

#### Notch Signaling Pathway

The Notch signaling pathway can promote macrophage differentiation into the M1 phenotype and contribute to the expression of proinflammatory cytokines (Castro et al., 2021; Chen et al., 2021). Notch proteins are proteolytically cleaved by furin-like proteinase at cleavage site 1 (S1) and processed into the mature form. Notch proteins consist of the Notch extracellular domain (NECD), the Notch intracellular domain (NICD), and a transmembrane domain. The interactions between the Notch proteins with Delta-like ligands (DLL1, DLL3, and DLL4) and the Jagged family (Jagged 1 and Jagged 2) induce the conformational changes in the receptors and initiate the Notch signaling pathway. Subsequent exposure to cleavage site 2 (S2) promotes proteolytic action by a tumor necrosis factor-α-convening enzyme (TACE), a member of the disintegrin and metalloprotease (ADAM) family, and the extracellular domain is released and taken in by adjacent cell with the ligand. Following cleavage by γ-secretase at S3, the intracellular NICD is activated and translocated into the nucleus. The activated NICD binds to the nuclear transcription factor CSL and dissociates from co-inhibitory receptors, promoting the transcription of the hairy and enhancer of split (HES) and hairy and enhancer of split-related with YRPW motif (HEY) family members and the inhibition of M2 macrophage polarization (Lin et al., 2018).

### Regulation of Macrophage Polarization by Inhibitory Receptors

**FIGURE 2:**
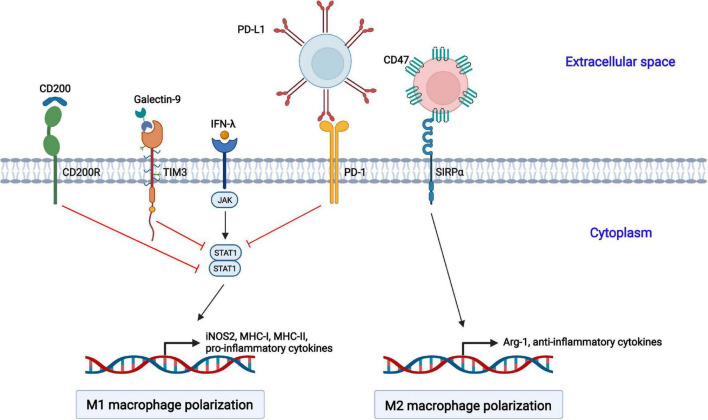
Negative regulation of macrophage polarization by cellular inhibitory receptors. The activation of CD200R, TIM3, and PD-1 suppresses STAT1-mediated macrophage polarization toward the M1 phenotype. CD47 interacts with SIRPα on the cell surface of macrophages and promotes macrophage polarization toward the M2 phenotype.

In addition to regulation by sophisticated signaling pathways, several inhibitory receptors are also involved in the polarization of macrophages (Figure 2).

#### Programmed Cell Death 1

Programmed cell death 1 (PD-1, also called CD279) is an inhibitory receptor and mainly expressed on immune cells, such as activated T and B lymphocytes, natural killer (NK) cells, dendritic cells (DCs), and macrophages (Cai et al., 2019). PD-1 is well studied in T lymphocytes. The interaction of PD-1 and its ligand PD-ligand 1 (PD-L1, also called B7-H1 or CD274) delivers negative regulatory signals and inhibits T cell activation through targeting the PI3K-Akt and Ras-MEK-ERK pathways (Boussiotis, 2016). Moreover, PD-1 plays a vital role in the function and phenotype of macrophages. PD-1 suppresses M1 polarization by decreasing the phosphorylation of STAT1 and promotes M2 polarization through increasing the phosphorylation of STAT6, which in turn reduces the expression of IL-12 (Ma et al., 2011; Yao et al., 2014).

#### T Cell Immunoglobulin and Mucin Domain-Containing Protein 3

T cell immunoglobulin and mucin domain-containing protein 3 (TIM3) was initially identified as a marker molecule on CD4^+^ and CD8^+^ T cells producing IFN-γ (Wolf et al., 2020). Subsequently, DCs, NK cells, macrophages, and mast cells were also shown to express TIM3 (Zhao et al., 2020). TIM3 consists of four distinct domains: a variable immunoglobulin domain (IgV), a mucin domain, a transmembrane domain, and an intracellular region (Ocaña-Guzman et al., 2016). TIM3 interacts with its ligand galectin-9, which leads to the phosphorylation of the intracellular tail by the Src and Tec family kinases, reducing the phosphorylation of STAT1 but augmenting that of STAT3. The activated TIM3/galectin-9 signaling pathway inhibits the LPSs-mediated polarization of macrophages toward the M1 phenotype (Zhang W. et al., 2019).

#### CD200 Receptor

The CD200 receptor (CD200R) belongs to the immunoglobulin superfamily of the mainly expressed on T cells and myeloid cells, and it contains one NH_2_-terminal extracellular domain, one transmembrane domain, and a short C-terminal intracytoplasmic domain (Ocaña-Guzman et al., 2018; Kotwica-Mojzych et al., 2021). The interaction of CD200R with its ligand CD200 results in the phosphorylation of tyrosine residues in the intracellular tail of CD200R by Src family kinases. Subsequently, Dok2 is recruited and binds to the phosphorylated tyrosine residues, and Ras-specific GTPase-activating protein (RasGAP) is further recruited to Dok2, inducing the inhibition of the Ras-ERK and PI3K kinases and the activation of STAT1.

#### CD47

CD47 is a member of the immunoglobulin superfamily (IgSF) and is widely expressed on hematopoietic and non-hematopoietic cells (Hayat et al., 2020). It contains a single N-terminal extracellular IgV domain, five transmembrane helices, and a C-terminal cytoplasmic tail. CD47 interacts with signal regulatory protein alpha (SIRPα) on macrophages, causes the tyrosine phosphorylation of SIRPα, and promotes the interaction with the phosphatase SHP-1, resulting in the transduction of “Don’t eat me” signals to macrophages and the induction of macrophage polarization into the M2 phenotype (Murata et al., 2014; Lin et al., 2018). CD47 is the best studied in antitumor treatment, and anti-CD47 antibody-based therapies targeting the CD47-SIRPα axis enhance the phagocytosis of cancer cells by macrophages (Zhang W. et al., 2020).

### Involvement of Cell Metabolism in Macrophage Polarization

Macrophage polarization is also coordinately regulated by metabolic networks, such as glucose, lipids, amino acids, and iron metabolism, described as “immunometabolism” (Van den Bossche et al., 2017; Yan and Horng, 2020; Xia et al., 2021). Glucose is principally metabolized through glycolysis, the pentose phosphate pathway (PPP), and the Krebs cycle (tricarboxylic acid cycle, TCA cycle) (Zhang Q. et al., 2021). Glucose metabolism may play an essential role in macrophage polarization. M1 macrophages are characterized by a high rate of glycolysis, and glycolysis produces adenosine triphosphate (ATP) and supplies glucose-6-phosphate to the PPP, promoting the generation of intermediates for amino acids, ribose, and NADPH that are required by inflammatory macrophages. In addition, the enzymes required for glycolysis promote proinflammatory M1-type macrophages (Van den Bossche et al., 2017). For instance, hexokinase 1 (HK1) positively regulates the NLRP3 inflammasome activation to produce IL-1β. In M2-polarized macrophages, oxidative phosphorylation (OXPHOS) and fatty acid oxidation (FAO) are increased to produce additional ATP, supporting the functions of these anti-inflammatory macrophages, such as tissue repair.

## Viral Infections Induce Macrophage Polarization

Macrophages are polarized into different phenotypes upon viral infections (Table 1). Some viruses induce macrophage polarization toward the M1 phenotype, while others promote M2 polarization. Moreover, several viruses cause complex polarization of macrophages depending on viral strains, infection stages, and gender of the host. Virus-infected macrophages are usually polarized into the proinflammatory M1 and anti-inflammatory M2 phenotypes in the early and late stages of infection, respectively (Burdo et al., 2015). Generally, pathogenic virus strains inhibit the antiviral responses of M1-polarized macrophages and skew macrophage polarization toward the M2 phenotype, whereas attenuated virus strains induce the M2 phenotype (Ferrer et al., 2019). The susceptibility of macrophages to different subtypes of influenza viruses (IVs) varies, and most of the H5N1 subtype highly pathogenic avian influenza viruses (HPAIVs) isolates can productively infect macrophages and induce M1 polarization (Marvin et al., 2017). Therefore, these studies suggests that the induction of M1 macrophage polarization is involved in clearance of invading viruses, but the severe injuries can be caused by hyperactivation or persistent activation characterized by robust proinflammatory cytokines. Therefore, the infections of virulent viruses usually results in illness or even death of hosts.

**TABLE 1:** Summarization of virus-induced macrophage polarization.

Virus	Family	Viral genome type	Polarized phenotype	References
Junin virus (JUNV)	*Arenaviridae*	−ssRNA	Attenuated Candid 1 strain: M1; Pathogenic P strain: M2	Ferrer et al., 2019
Influenza virus (IV)	*Orthomyxoviridae*	Segmented −ssRNA	M1	Zhang et al., 2018
Severe fever with thrombocytopenia syndrome virus (SFTSV)	*Bunyaviridae*	Segmented −ssRNA	M2	Zhang L. et al., 2019
Foot-and-mouth disease virus (FMDV)	*Picornaviridae*	+ssRNA	M1	Sebastian et al., 2020
Coxsackievirus B3 (CVB3)	*Picornaviridae*	+ssRNA	Male: M1; Female: M2	Li et al., 2009
Porcine reproductive and respiratory syndrome virus (PRRSV)	*Arteriviridae*	+ssRNA	M2	Wang et al., 2017
West Nile virus (WNV)	*Flaviviridae*	+ssRNA	M1	Stone et al., 2019
Hepatitis C virus (HCV)	*Flaviviridae*	+ssRNA	M2	Bility et al., 2016
Severe acute respiratory syndrome coronavirus 2 (SARS-CoV-2)	*Coronaviridae*	+ssRNA	M2	Boumaza et al., 2021
Human immunodeficiency virus type 1 (HIV-1)	*Retroviridae*	+ssRNA	Acute phase: M1; Chronic phase: M2	Burdo et al., 2015
Hepatitis B virus (HBV)	*Hepadnaviridae*	cccDNA	M2	Bility et al., 2014
Epstein-Barr virus (EBV)	*Herpesviridae*	dsDNA	M2	Zhang B. et al., 2020
Human cytomegalovirus (HCMV)	*Herpesviridae*	dsDNA	M1	Chan et al., 2008
African swine fever virus (ASFV)	*Asfarviridae*	dsDNA	M1	Tatoyan et al., 2020

## M1-Polarized Macrophages Combat Viral Infection Through Multiple Antiviral Strategies

Activated macrophages, mainly M1-polarized macrophages, play essential roles in fighting against viral infections through multiple strategies, including producing an oxidized environment and antiviral cytokines or activating other immune cells.

### Production of Reactive Species

Reactive species (RS) produced in M1 macrophages include reactive oxygen species (ROS) and reactive nitrogen species (RNS), which lead to a highly oxidative environment (Molteni et al., 2014; Camini et al., 2017). ROS, mainly hydrogen peroxide (H_2_O_2_), superoxide anions (⋅O_2_-), and hydroxyl radicals (⋅OH), are generated by mitochondria, NADPH oxidase (NOX), endoplasmic reticulum (ER), and/or peroxisomes (Rendra et al., 2019). Furthermore, nitric oxide (NO) is the most important RNS in macrophages and is synthesized through the conversion of L-arginine by iNOS2 (Uehara et al., 2015). NO exerts potent antimicrobial activity with a broad spectrum *via* different mechanisms. NO suppresses vaccinia virus (VACV) replication by impairing viral ribonucleotide reductase activity (Fujikura et al., 2009). Similarly, NO exerts an inhibitory effect on severe acute respiratory syndrome coronavirus 2 (SARS-CoV-2) replication by targeting the activity of the 3CL protease (Akaberi et al., 2020). Clinically, inhaled NO has been demonstrated to be an effective therapeutic agent against SARS-CoV-2 infection and severe pulmonary consequences (Adusumilli et al., 2020; Ricciardolo et al., 2020). However, the expression level of iNOS2 varies in macrophages of different species, resulting in differences in NO production (Schneemann and Schoedon, 2002; Zelnickova et al., 2008). Rat pulmonary alveolar macrophages (PAMs) produce a large amount of NO, and bovine PAMs produce a relatively low level of NO using L-arginine as substrate, while caprine, lapine, and porcine PAMs do not produce NO. Thus, NO may be a potential host range factor that restricts cross-species transmission.

### Secretion of Antiviral Cytokines

M1 macrophages are characterized by the robust production of proinflammatory cytokines, such as TNF-α, IL-1, IL-6, IL-8, and IL-12, which exert antiviral activities directly or indirectly (Arango Duque and Descoteaux, 2014). The replication of influenza A virus (IAV), human immunodeficiency virus type 1 (HIV-1), porcine respiratory and reproductive syndrome virus (PRRSV), classical swine fever virus (CSFV), and mouse adenovirus is inhibited by the direct treatment of cells with TNF-α (Van Campen, 1994; Lane et al., 1999; Li et al., 2015; Li et al., 2016; Pant et al., 2020). The mechanisms by which TNF-α suppresses viral infections may vary in different viruses. TNF-α exhibits an inhibitory effect on HIV-1 by inducing the production of RANTES and decreasing the expression of the C-C chemokine receptor (CCR5), which can serve as a co-receptor for HIV-1 entry (Lane et al., 1999). Moreover, TNF-α inhibits CSFV replication through the NF-κB signaling pathway and the induction of IRF1-dependent type I IFN responses (Liniger et al., 2021). IL-6 exhibits potent antiviral effects on hepatitis B virus (HBV) and varicella-zoster virus replication (Kuo et al., 2009; Como et al., 2018). Mechanistically, IL-6 inhibits HBV infection through preventing the formation of genome-containing nucleocapsids and accumulation of the HBV cccDNA. In contrast, antiviral activity of IL-8 is rarely documented, which exhibits an inhibitory effect on HIV-1 replication through suppression of viral transcription (Csoma et al., 2006). IL-1β synergizes with IFN-α to suppress HCV replication by negatively regulating ERK activation (Guo et al., 2020). In addition, IL-12 promotes the differentiation of naïve CD4^+^ T cells into Th1 cells and activates NK cells to fight against the viral infections (Wang et al., 2012).

### Activating Other Immune Cells

In M1-polarized macrophages, B7 family molecules (including CD80 and 86) and MHC molecules are expressed at relatively high levels, which is required for T cell activation and downstream antiviral responses (Guerriero, 2019). In addition, macrophage-derived cytokines, including IL-2 and IL-12, promote the further activation of T cells and corresponding antiviral responses. NK cells also play an essential role in antiviral immune responses by secreting of perforin or inducing of death receptor-mediated apoptosis (Zwirner et al., 2021). M1 macrophages, but not M2 macrophages, enhance the cytotoxicity of NK cells in an IL-1β-, IFN-β-, or IL-15-dependent fashion (Mattiola et al., 2015).

## Macrophages Are Exploited as Efficient Vehicles for Viral Replication and Dissemination

The host cells with a high susceptibility to viral infections usually have a sufficient lifespan without apoptosis or robust antiviral responses. However, macrophages do not possess these characteristics. Many viruses still exploit macrophages as vehicles for efficient infection (Nikitina et al., 2018). In addition to the pivotal roles in the regulatory network of immune cells, macrophages promote viral infections at entry stage, deliver viruses to permissive tissues and provide an immunofavorable microenvironment.

### Promoting Efficient Viral Infection at Entry Stage

Macrophages are the first line of defense against viral invasion, increasing the possibility of exposure to viruses (Mercer and Greber, 2013). In addition, macrophages are professional antigen-presenting cells (APCs) that constitutively undergo macropinocytosis and phagocytosis. Thus, in addition to receptor-mediated endocytosis, viruses can be captured passively, facilitating viral entry into the cells through multiple routes. African swine fever virus (ASFV) is a large DNA virus that primarily infects macrophages (Gaudreault et al., 2020; Masujin et al., 2021). ASFV hijacks both clathrin-mediated endocytosis and macropinocytosis to invade macrophages, and the exploitation of multiple endocytic routes markedly increases the efficiency of ASFV entry (Sánchez et al., 2017).

### Delivering Viruses to Permissive Tissues/Organs

Macrophages circulate in almost all tissues in the bloodstream and are differentiated into tissue-resident macrophages, executing tissue-specific functions. Therefore, viruses in macrophages are delivered to the permissive tissues, promoting further viral infection. Moreover, macrophages interact with different cell populations, and thus viruses can be disseminated through direct cell-to-cell contact (Nikitina et al., 2018).

### Providing a Suitable Extracellular Microenvironment

Due to the essential roles of macrophages in regulating the activity of other immune cells (such as T cells and NK cells), as described in section “Activating Other Immune Cells,” viruses may evolve to exploit macrophages as their main target cells and suppress the direct and indirect antiviral effects from mainly T cells and NK cells by negatively regulating macrophage polarization, thus promoting viral infections in an immunofavorable microenvironment.

## Viruses Have Evolved Multiple Strategies to Counteract M1 Phenotype Macrophages

**FIGURE 3:**
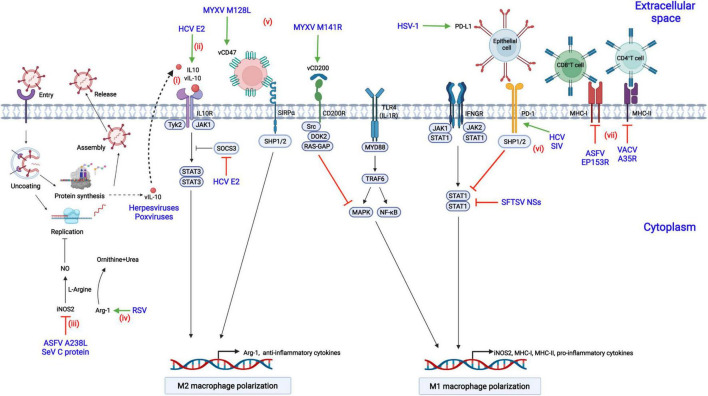
Modulation of macrophage polarization by viral proteins. (i) Herpesviruses or poxviruses encode the homolog of IL-10 (vIL-10), which binds to IL-10R, triggers the activation of STAT3, and contributes to M2 macrophage polarization; (ii) Hepatitis C virus (HCV) encodes the E2 protein, which upregulates IL-10 production by increasing the phosphorylation of STAT3 and reducing the expression of SOCS3; (iii) African swine fever virus (ASFV) infection results in the suppression of nitrogen oxide (NO) production by downregulating inducible nitric oxide synthase 2 (iNOS2) *via* the A238L protein; (iv) Respiratory syncytial virus (RSV) inhibits NO production by decreasing the expression of arginase 1 (Arg-1) and the level of L-arginine; (v) Myxoma virus (MYXV) encodes the M141R (the viral CD200 homolog) and M128L (the viral CD47 homolog) proteins, which suppress the M1 phenotype polarization of macrophages; (vi) Acute infection with herpes simplex virus type 1 (HSV-1) and chronic infection with HCV or simian immunodeficiency virus (SIV) lead to the increased expression of PD-L1 or PD-1, inhibiting STAT1 activation and M1 macrophage polarization; (vii) The vaccinia virus (VACV) A35R protein suppresses the antiviral responses of T cells by negatively regulating the major histocompatibility complex class II (MHC-II) antigen presentation, while the ASFV EP153R and the HIV-1 Nef proteins downregulate MHC-I expression.

Viruses have evolved diverse strategies to evade the antiviral responses of M1-polarized macrophages to achieve a suitable cellular environment for replication in macrophages, including inhibition of M1 macrophage polarization or antagonization of downstream antiviral responses (Figure 3).

### Induction of Macrophage Polarization to the M2 Phenotype by Regulating the Activity of Inhibitory Receptors

Viral infection promotes macrophage polarization toward the M2 phenotype by increasing the expression of cellular or viral inhibitory receptors, including PD-1, PD-L1, and the viral homologs of CD200 and CD47.

#### Programmed Cell Death 1 and Its Ligand PD-L1

In chronic HCV infection, the expression of PD-1 is upregulated, and the production of IL-12 and the activation of STAT1 are suppressed in macrophages (Ma et al., 2011). Similarly, in chronic HIV or Simian immunodeficiency virus (SIV) infection of rhesus macaques, the expression of PD-1 is increased in alveolar macrophages, and the expression of proinflammatory cytokines is dramatically decreased compared with that in naïve macaques, suggesting that M2 polarization are promoted (Burdo et al., 2015; Hunegnaw et al., 2019). PD-L1 is constitutively expressed in the corneal epithelium and is up-regulated upon herpesvirus type 1 (HSV-1) infection (Jeon et al., 2018). Knockout of PD-L1 increases the migration of inflammatory cells into viral lesions and decreases virus titers due to the impaired viral clearance by macrophages.

#### Viral CD200 Homolog

Myxoma virus (MYXV) encodes the *M141R* gene, a homolog of CD200. It interacts with CD200R and inhibits M1 macrophage polarization in an NF-κB-dependent fashion (Zhang et al., 2009). Although M141R is not essential for MYXV replication in macrophages, the M141R-deletion MYXV mutant is highly attenuated in rabbits with high-level IFN-γ, suggesting that M141R affects virus pathogenicity by skewing macrophage polarization toward the M2 phenotype (Cameron et al., 2005). Similarly, human herpesvirus 8 (HHV-8) encodes the K14 protein, similar to CD200, and may skew macrophage polarization toward the M2 phenotype by interacting with CD200R (Foster-Cuevas et al., 2004).

#### Viral CD47 Homolog

The *M128L* gene, encoded by MYXV, is a five-span transmembrane protein similar to CD47. M128L is not essential for viral replication *in vitro*, but the M128L gene-deleted virus is significantly attenuated in rabbits, suggesting that CD47-like M128L is a virulence factor of MYXV (Cameron et al., 2005). Mechanistically, the knockout of M128L from the viral genome may contribute to M1 macrophage polarization, increasing the expression level of iNOS2 and mounting robust antiviral responses.

### Interference With Macrophage Polarization-Associated Signaling Pathways

STAT3 and its induction of downstream IL-10 are critical for macrophage polarization to the M2 phenotype, while STAT1 is necessary for M1 polarization (Wilson, 2014). The hepatitis C virus E2 protein increases IL-10 expression in macrophages, promoting macrophage polarization toward the M2 phenotype (Kwon et al., 2019). Mechanistically, the E2 protein inhibits the activation of STAT1 and increases the phosphorylation of STAT3 through reducing the phosphorylation level of SOCS3, thus promoting the transcription of IL-10 and M2 macrophage polarization. Similarly, the infection of severe fever with thrombocytopenia syndrome virus (SFTSV) drives macrophage polarization toward the M2 phenotype to facilitate its efficient replication through upregulating miR-146b by the nonstructural protein encoded by the S segment (NSs). miR-146b functions by inhibiting the expression and phosphorylation of STAT1, inducing the macrophage differentiation to the M2 phenotype (Zhang L. et al., 2019). In addition, some viruses, including poxviruses and herpesviruses, encode functional viral IL-10 (vIL-10) and enhance viral infections by directly shifting macrophages polarization toward the M2 phenotype (Ouyang et al., 2014).

### Inhibition of Nitric Oxide Production in Macrophages

NO is a prominent antiviral effector in M1-polarized macrophages through a variety of mechanisms that have been described in section “Production of Reactive Species.” Respiratory syncytial virus (RSV) infection results in the increased level of NO that which interferes with viral replication (Kao et al., 2001). However, this antiviral activity can be antagonized by RSV by inducing the constitutive expression of Arg-1, hydrolyzing L-arginine to L-ornithine and urea and suppressing the reactants required for the synthesis of NO (Santiago-Olivares et al., 2019). NO is mainly produced by iNOS2 in macrophages. ASFV A238L is an NF-κB and NFAT inhibitor and suppresses NO production by inhibiting iNOS2 transcription (Granja et al., 2006). Similarly, SeV infection also results in the suppression of NO production through the downregulation of the expression of iNOS2 (Odkhuu et al., 2014). Mechanistically, the C protein of SeV blocks the activation of the JAK-STAT signaling pathway, resulting in the inhibition of M1 macrophage polarization and thus iNOS2 transcription.

### Decreasing the Production of Proinflammatory Cytokines

Activated macrophages produce high-level proinflammatory cytokines upon viral infections. The glycoprotein (GP) of Ebola virus (EBOV) can be cleaved by cellular TACE into secreted GP (sGP) (Zhu et al., 2019). Treatment of activated macrophages with sGP inhibits the production of proinflammatory cytokines, such as TNF-α and IL-6 (Bradley et al., 2018). Moreover, the migratory ability of macrophages is impaired by sGP due to the decreased expression of CD11b. Similarly, Rift Valley fever virus (RVFV) productively infects macrophages in humans and suppresses the expression of proinflammatory cytokines (including IFN-α2, IFN-β, and TNF-α) by encoding the NSs protein (McElroy and Nichol, 2012).

### Suppression of Antigen Presentation by Macrophages

Antigen presentation by macrophages is critical for activating T lymphocytes to clear invading viruses, but viruses have developed multiple immune evasion strategies to counteract the antiviral effects. HIV-1 escapes from the killing by CD8^+^ cytotoxic T lymphocytes (CTLs) by encoding the Nef and Gag proteins (Hendricks et al., 2021). Among them, Nef is associated with the regulation of the antigen-presenting ability of macrophages. More specifically, the Nef protein promotes the degradation of MHC-I, reducing its expression of on the cell surface (Schaefer et al., 2008). VACV infection inhibits T-cell priming by decreasing the expression of MHC-II on the cell surface of APCs and the subsequent synthesis of chemokines and cytokines (Rehm et al., 2009). Further study demonstrated that the VACV A35R protein is involved in this inhibition. Mechanistically, the A35R protein is localized to endosomes and may impair the processing and presentation of MHC-II-restricted antigens (Rehm et al., 2010). Similarly, the ASFV EP153R protein, which contain a lectin-like domain, decreases the expression of MHC-I antigens on the cell surface and interferes with the exocytosis and presentation of antigens in association with MHC-I on the cell surface through its lectin domain, which may result in the inhibition of antiviral responses by CTLs (Hurtado et al., 2011).

Besides these strategies, there may be more evasion mechanisms that remain to be uncover. For example, large DNA viruses (such as poxviruses and herpesviruses) may encode several soluble viral proteins to block the MHC molecules even in large abundance on the cell surface to suppress antigen presentation. Furthermore, several viral proteins may be involved in the regulation of glucose metabolism and suppress the antiviral responses of M1-polarized macrophages by negatively regulating the glycolysis pathway.

## Exploitation of M2 Macrophage Polarization for Efficient Infection

In response to the robust antiviral responses induced by M1-polarized macrophages, several viruses employ multiple immune escape strategies to overcome these host defenses, while other viruses evolve to skew macrophages to the M2 phenotype (Table 1). The M2-polarized macrophages are characterized by increased expression of marker molecules or impaired antiviral responses, which promote primary or secondary infections.

### Exploitation of Surface Markers on M2 Macrophages for Virus Entry

PRRSV mainly replicates in porcine PAMs, resulting in the polarization of PAMs toward the M2 phenotype characterized by the high-level expression of CD163, which serves as a functional receptor for virus entry (Wang et al., 2017; Su et al., 2021). The viral protein(s) essential for regulating macrophage polarization remain(s) unknown, but a study has been implied that PRRSV induces M2-polarized macrophages for efficient growth while simultaneously counteracting the antiviral responses of M1-polarized macrophages (Wang et al., 2017). Similarly, treatment of macrophages with IL-4/IL-13 enhanced the infection of the recombinant vesicular stomatitis virus encoding the EBOV-GP (rVSV/EBOV-GP), and a further study showed that the macrophages were polarized toward the M2a phenotype by IL-4/IL-13 with increased expression of specific intercellular adhesion molecule-3-grabbing non-integrin related gene 3 (SIGNR3), promoting the entry of rVSV/EBOV-GP into the host cells (Rogers et al., 2019).

### Enhancement of Co-infections With Viruses and Bacteria

Viral and bacterial co-infections are often observed in infectious diseases in humans and animals and contribute to the deterioration of the illness, but the underlying mechanisms remain to be investigated. The modulation of macrophage polarization by viruses may play an important role in augmenting infections. RSV infection promotes M2-like macrophage polarization by increasing the expression of growth arrest-specific 6 (Gas6), which interacts with Axl and suppresses the antibacterial responses of macrophages (Shibata et al., 2020). Thus, RSV-infected patients are more susceptible to subsequent pneumococcal infections, which triggers secondary pneumococcal pneumonia. Moreover, the reduced production of IL-18 impairs the antibacterial activity of NK cells and then suppresses the production of IFN-γ, NO, and TNF-α. In contrast, the capsid protein (Cap) of porcine circovirus type 2 (PCV2) suppresses the expression of interferon regulatory factor 4 (IRF4) by inhibiting the transcription of jumonji domain-containing 3 (JMJD3), thus promoting M1 macrophage polarization and bacterial engulfment (Zhang W. et al., 2021).

In addition to the promotion of bacterial infection by viruses, it is very likely that the infection with one virus can be enhanced by another virus through regulation of macrophage polarization. Therefore, elucidating the mechanisms that mediate secondary viral infections may provide clues to screen functional receptors for virus entry and antiviral drugs.

## Treatments of Viral Diseases by Targeting Macrophage Polarization

M1-polarized macrophages play essential roles in fighting against viral infections. Therefore, chemical reagents or natural compounds that promote macrophage polarization into the M1 phenotype may be utilized for antiviral treatment. For instance, baicalin (a natural compound) inhibits IAV infection by inducing antiviral M1 macrophages and activating the IFN signaling pathway (Li and Wang, 2019; Geng et al., 2020). However, the hyperactivation of macrophages, known as macrophage activation syndrome (MAS), causes aberrant inflammatory responses (or “cytokine storm”) and severe illness to the hosts (Mahmudpour et al., 2020). SARS-CoV-2 infection causes excessive inflammation in the lungs and progresses to acute respiratory distress syndrome (ARDS) (Otsuka and Seino, 2020). Thus, anti-inflammatory therapeutics can be used to treat coronavirus disease 2019 (COVID-19) (Napoli et al., 2021). To date, IL-1R antagonists (IL-1RA), IL-6R antagonists (IL-6RA), or anti-inflammatory drugs (tofacitinib) have been used in clinical practice. Regarding the viral diseases engaged by M2 macrophages, the treatments of skewing macrophage polarization to the M1 phenotype or blocking several marker molecules that are utilized by viruses for infection will be potential strategies in the future.

## Concluding Remarks and Prospects

In the sophisticated immune system, macrophages are the first line of defense against infection and play important roles in the clearance of viruses, bacteria, and solid tumor cells. Macrophages are polarized into the proinflammatory (M1) or anti-inflammatory (M2) phenotype upon exposure to diverse stimuli. M1 macrophages elicit robust antiviral responses by expressing high-level proinflammatory cytokines, promoting cellular oxidation, and/or inducing the activation of other immune cells. To replicate in macrophages efficiently, viruses have employed various strategies to counteract the antiviral responses elicited by M1 macrophages or to skew macrophage polarization to favor the M2 phenotype associated with impaired immune responses. Moreover, some viruses have evolved to adapt to polarized macrophages and exploit marker molecules for efficient replication.

Despite the importance of macrophage polarization in viral infections, the regulatory mechanisms by viruses, which may be the basis for developing therapies against viral diseases, remain to be further elucidated. The worldwide-spread influenza (in 1918, 1957, 1968, and 2009) and coronavirus (in 2003, 2015, and 2019) pandemics showed that the hyperactivated macrophages-mediated “cytokine storm” can cause severe illness (Tisoncik et al., 2012; Liu et al., 2016; Ryabkova et al., 2021). Elucidating the regulatory mechanisms through which viruses regulate macrophage polarization will contribute to the discovery of therapeutic targets for treating viral diseases.

## Author Contributions

SY designed the review and drafted the manuscript. H-JQ and SL supervised the review and made critical revisions to the manuscript. HG edited the manuscript. All authors contributed to the article and approved the submitted version.

## Conflict of Interest

The authors declare that the research was conducted in the absence of any commercial or financial relationships that could be construed as a potential conflict of interest.

## Publisher’s Note

All claims expressed in this article are solely those of the authors and do not necessarily represent those of their affiliated organizations, or those of the publisher, the editors and the reviewers. Any product that may be evaluated in this article, or claim that may be made by its manufacturer, is not guaranteed or endorsed by the publisher.
